# Gastric Intestinal Metaplasia: Challenges and the Opportunity for Precision Prevention

**DOI:** 10.3390/cancers15153913

**Published:** 2023-08-01

**Authors:** Douglas Tjandra, Rita A. Busuttil, Alex Boussioutas

**Affiliations:** 1 Central Clinical School, Monash University, 99 Commercial Rd, Melbourne, VIC 3004, Australia; rita.busuttil@monash.edu; 2 Department of Gastroenterology, The Alfred Hospital, 55 Commercial Rd, Melbourne, VIC 3004, Australia

**Keywords:** premalignant, intestinal metaplasia, gastric cancer, cancer surveillance

## Abstract

**Simple Summary:**

Gastric adenocarcinoma is the fifth most common cancer worldwide and the fourth most lethal. It is often asymptomatic at an early stage, when survival rates are highest (>90%) with minimally invasive endoscopic intervention or surgery. Gastric intestinal metaplasia (GIM) is a persistent, premalignant lesion in the stomach, arising in the context of chronic inflammation, which predisposes one to gastric cancer. It is considered a pivotal stage along a continuum to gastric cancer and has a median latency period of ~6 years before progression to cancer typically occurs, offering a window of opportunity for intervention. However, only a small proportion (0.25–2.5%) with GIM ultimately progress to cancer; therefore, approaches to surveillance vary widely around the world. We summarise the current evidence supporting best clinical practice in the diagnosis, assessment and management of GIM, and the opportunities to achieve precision in predictions in the coming decades.

**Abstract:**

GIM is a persistent, premalignant lesion whereby gastric mucosa is replaced by metaplastic mucosa resembling intestinal tissue, arising in the setting of chronic inflammation, particularly in the context of *Helicobacter pylori*. While the overall rates of progression to gastric adenocarcinoma are low, estimated at from 0.25 to 2.5%, there are features that confer a much higher risk and warrant follow-up. In this review, we collate and summarise the current knowledge regarding the pathogenesis of GIM, and the clinical, endoscopic and histologic risk factors for cancer. We examine the current state-of-practice with regard to the diagnosis and management of GIM, which varies widely in the published guidelines and in practice. We consider the emerging evidence in population studies, artificial intelligence and molecular markers, which will guide future models of care. The ultimate goal is to increase the detection of early gastric dysplasia/neoplasia that can be cured while avoiding unnecessary surveillance in very low-risk individuals.

## 1. Introduction

Gastric intestinal metaplasia (GIM) is a premalignant state predisposing to gastric adenocarcinoma, a devastating and often lethal diagnosis. However, only a small proportion of those with GIM develop cancer, and predicting progression has remained a major clinical challenge. In this review, we outline the clinicopathological factors that are known to increase the risk of progression to cancer. We reflect on the emerging directions of management, particularly the increasing understanding of the molecular landscape of GIM and gastric adenocarcinoma, which will be important in improving the precision of risk stratification and optimising models of care.

## 2. Background

### 2.1. Global Burden of Gastric Cancer

Despite reducing incidence, gastric adenocarcinoma remains the fifth most common cancer worldwide, and the fourth most lethal [1]. In 2020, there were an estimated 1,089,103 new cases (5.6% of all new cancers) and 768,793 deaths (7.7% of all cancer-related deaths), which are projected to increase to ~1.8 million new cases and ~1.3 million deaths by 2040 [1,2]. The highest incidence is found in older males, although increasing rates amongst the younger patients/age group (<50 years) have been reported in recent years [3,4]. There is significant geographical variation, with higher risk in Eastern Asia (Korea, Japan, Mongolia, China), Eastern Europe, and Central and South America compared with Northern America, Northern Europe, Africa and Australia [3].

Geographic risk generally correlates with the rate of infection with the Gram-negative, spiral bacterium *Helicobacter pylori* [5]. It is now well-recognised as a class I carcinogen [6] and ~50% of the world’s population are chronically infected in early life. *H. pylori* is considered the predominant cause (90%) of non-cardia (lower stomach) gastric cancers, which is the most common form globally, and economic development, improvements in sanitation, and possibly the increased use of antibiotics have most likely led to the reduction in worldwide incidence seen in recent decades, rather than significant advances in secondary prevention or treatment [7,8]. Exceptions to this association have been identified, the so-called ‘African enigma’, whereby some countries with a high prevalence of *H. pylori* infection have low rates of gastric cancer and other upper gastrointestinal tract disease; multiple potential explanations have been proposed, including differing strains of *H. pylori*, concurrent helminthic infection modulating the immune response, other environmental exposures, and under-diagnosis in settings of socioeconomic inequality [9,10,11]. Additionally, there are other biologic pathways to gastric adenocarcinoma, particularly the cardia (upper stomach) type, where gastro-oesophageal reflux may play a major role in causing a cancer that resembles oesophageal adenocarcinoma [12].

Several paradigms have been proposed to understand the different pathophysiological pathways to gastric adenocarcinoma. Histologically, the Lauren classification is widely used to divide cancers into two main types: intestinal and diffuse [13]. Intestinal-type gastric cancer tends to be more well-differentiated, occurs in older males with *H. pylori* infection, and has a better prognosis than the diffuse-type, which has more poorly differentiated and non-cohesive cells infiltrating the wall, is seen more frequently in younger females, and is sometimes associated with hereditary mutations in E-Cadherin (*CDH1*) [14,15]. The Cancer Genome Atlas Research Network used 295 gastric cancers to characterise four molecular subtypes to guide a better understanding of the varied cancer biologies: a chromosomal instability subtype (enriched with intestinal-type tumours), a genomically stable subtype (enriched with diffuse-type tumours), an Epstein–Barr virus-positive subtype, and a microsatellite unstable subtype [16].

Despite the reduction in global incidence and increasing understanding of the molecular characteristics, the prognosis of gastric cancer remains poor, as it is usually asymptomatic until it reaches an advanced stage, and the majority of cases, therefore, present late. The median survival of T3 or T4 stage cancers is from 3 to 5 months without treatment, or from 6 to 14 months with palliative chemotherapy [17]. By contrast, early gastric adenocarcinoma has a 5-year survival rate of >90% [18], and early detection improves outcomes, as seen in South Korea and Japan, where population-based screening increased 5-year survival to 60–70%, as compared with <40% 3-year survival in Western countries [19,20,21,22]. Their guidelines recommend that asymptomatic adults from 40 to 75 years of age in South Korea and adults aged ≥50 years in Japan undergo biannual gastric cancer screening using upper endoscopy [23,24]. However, it would not be practical to perform general population-based screening in low-incidence countries; therefore, identifying an alternative risk-stratification strategy would be of use.

### 2.2. Gastric Intestinal Metaplasia as a Premalignant State

Correa, in his seminal paper in 1975, proposed a model of stepwise histologic progression from normal gastric mucosa to gastritis, chronic atrophic gastritis, gastric intestinal metaplasia, from low- to high-grade dysplasia, and, finally, intestinal-type gastric cancer occurring in the setting of chronic inflammation [25]. The model was later updated to include the crucial role of *H. pylori* as an initiator of inflammation [26,27]. It remains unclear whether the same pathway can lead to diffuse-type gastric cancer, although both *H. pylori* and various precursor lesions in the Correa model have been shown to be in association with diffuse-type gastric cancers [28,29].

GIM, the stage preceding dysplasia in the Correa model, is a persistent, premalignant state characterised by the replacement of epithelial cells in the gastric mucosa with epithelial cell types found in the intestine. GIM is itself not often the cause of any symptoms and is usually an incidental finding on upper endoscopy for other indications.

Similar to gastric cancer, incidence of GIM mirrors the prevalence of *H. pylori* in the community, occurring in up to 25% in high-incidence countries and 5% in low-incidence countries [30]. IM may, therefore, be a form of adaptation by the gastric stem cell to protect against chronic infection with *H. pylori* and promote healing [31]. *H. pylori* is able to evade and manipulate both innate and adaptive immune defenses by utilising an arsenal of microbial enzymes and virulence factors to establish chronic infection at the level of the gastric mucous layer and epithelial surface, although only some patients develop disease, suggestive of interactions with host genetic factors and possibly dysbiosis. Chronic *H. pylori* gastritis has been associated with increased myeloid cell activation, proinflammatory cytokines such as IL1β, IL10, IL8, IL17 and TNFα, skewed Th1/Th17 response and a specific IgG/IgA antibody response; polymorphisms in a number of these components appear to predispose to clinical disease [32,33,34,35].

Duodeno-gastric reflux with bile acids is another proposed mechanism of mucosal insult (analogous to gastric acid reflux in Barrett’s oesophagus) [36]. In both these causes of GIM, the initial pathogenic process is thought to occur in the gastric antrum, and this is where IM is most often initially seen, before progressing proximally towards the corpus and fundus, often along the lesser curve of the stomach (the so-called ‘Magenstrasse’); more extensive IM is considered a more advanced disease process [37]. In contrast, atrophy and intestinal metaplasia related to autoimmune gastritis, whereby T lymphocytes and autoantibodies target parietal cells, starts in the corpus where the parietal cells are located [38].

**Figure 1:**
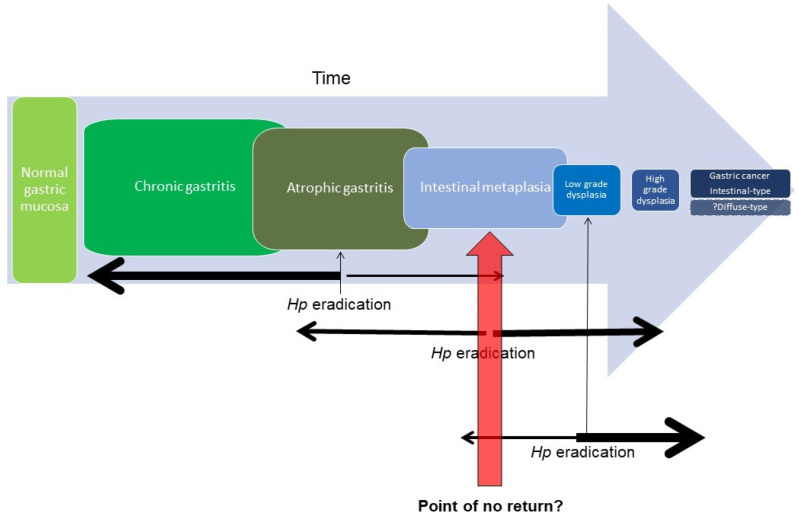
Schematic of progression along Correa cascade. Only a proportion will progress to the next stage, but with a decreasing latency period. The efficacy of *H. pylori* eradication may reduce with each subsequent step along the cascade due to accumulating genetic aberrations in the gastric epithelial stem cell.

The development of IM is considered a pivotal stage in gastric cancer progression, and it has been theorised that there may be a ‘point of no return’ at some stage along its biologic continuum. *H. pylori* eradication appears to be more effective for preventing gastric cancer before IM develops (Figure 1) [39,40], suggesting that irreversible genomic changes may occur in the gastric epithelial stem cell in the IM state. Clonality studies have demonstrated that metaplastic glands derive from the same clone through gland fission, and can be genetically related to dysplastic glands, further supporting the concept of the gradual acquisition of genetic changes until a final dysplasia-initiating event occurs [41]. Some studies have suggested that a subset of patients with IM may still regress with eradication of *H. pylori* [42,43], although assessment for histologic regression is limited by the potential for false-negative sampling errors, whereby residual foci of IM can be missed on biopsy. Nonetheless, such results reinforce the need for the early eradication of *H. pylori* to most effectively reduce the risk of progression. Even in established IM, *H. pylori* eradication appears to slow progression along the Correa cascade [44,45].

IM has been shown in meta-analysis to increase the risk of gastric cancer by 3.6-fold [37]. The median time to progression from IM is 6.1 years [46], and this premalignant latency period offers a window of opportunity for identification and preventative intervention in high-risk individuals, such as close monitoring for the development of dysplasia, which can be endoscopically managed with minimal invasion [46]. This can be compared with the relatively short latency period of dysplasia to cancer (2.6 years from low-grade dysplasia to cancer), and almost all patients proceed to gastric cancer once high-grade dysplasia develops [47]. The challenge is to determine which patients with GIM will progress, with rates of progression to cancer reported to be between 0.25 and 2.5% [37,48,49].

## 3. Predictors of Progression of Gastric IM

### 3.1. Clinical Features

In addition to *H. pylori* status, the two clinical factors that appear to increase the risk of progression of GIM to malignancy are age >50 years (hazard ratio 8.8, 95% CI 1.2–68.5 [50]) and family history of gastric cancer in a first-degree relative (hazard ratio 4.5, 95% CI 1.3–15.5 [51]). A detailed family history is always required to assess for underlying hereditary cancer syndromes. Other demographic and environmental risk factors implicated in a higher risk of gastric cancer are variably linked to an elevated risk of progression in those with GIM, including ethnicity/country of origin, smoking status, alcohol intake, concurrent autoimmune gastritis, high salt- or nitrosamine-containing diet, low intake of fresh fruit and vegetables, low exercise and obesity [31,51,52,53]. Certain exposures may increase risk in certain subgroups of IM; one prospective study found that smoking increases the risk of malignancy only amongst those with intermediate- or later-stage IM (based on histological scoring systems) [53].

### 3.2. Endoscopic Features

The major aims of high-quality upper endoscopy are to identify regions of GIM, determine the extent of IM and detect dysplasia or cancers. The extent of IM is defined as ‘limited’ (involving only one region of the stomach, antrum/incisura or body) or ‘extensive’ (extending beyond one region), with extensive IM conferring a 2-fold increased risk of progression compared with limited IM [51,54]. As outlined previously, dysplasia is the penultimate step in the Correa cascade before cancer, and identifying the visible foci of dysplasia allows for endoscopic intervention. Further emphasising the need for meticulous examination, a recent meta-analysis has demonstrated that ~10% of upper gastrointestinal cancers are missed at endoscopy within 3 years of diagnosis [55].

To optimise upper endoscopy, a mean procedure time of 7 min or longer has been associated with a significantly higher detection rate for dysplasia or neoplasia (odds ratio 3.42, 95% CI 1.25–10.38) [56]. A small volume of simethicone solution ± N-acetylcysteine, administered 31–60 min before upper endoscopy, can help to attain optimal mucosal visibility [57,58,59,60]. Detailed photo-documentation of all major anatomical landmarks from multiple angles should be routine (pylorus, antrum, incisura, transition zone, lesser and greater curves, fundus and cardia). Practices vary around the world but groups in Asia, where population-based endoscopic screening is performed, have proposed systematic screening protocols with at least 20–22 photographs to photo-document a standard screening gastroscopy [61].

Endoscopic changes consistent with different stages along the Correa cascade are readily distinguishable on careful examination. Atrophic gastritis is associated with a marginal turbid band (MTB), an enclosing whiteish line on the epithelial surface. GIM is also associated with MTB, and as it becomes more severe, a light-blue crest (LBC) also develops, which is a fine blue–white line on the crest of the epithelial surface. MTB is highly sensitive for GIM, while LBC is highly specific; a prospective study of 47 patients in South Korea found that the sensitivity, specificity and accuracy for MTB and LBC in GIM were 100%/66%/81.7% and 72.1%/96.0%/84.9%, respectively, using histology as the gold-standard [62].

**Figure 2:**
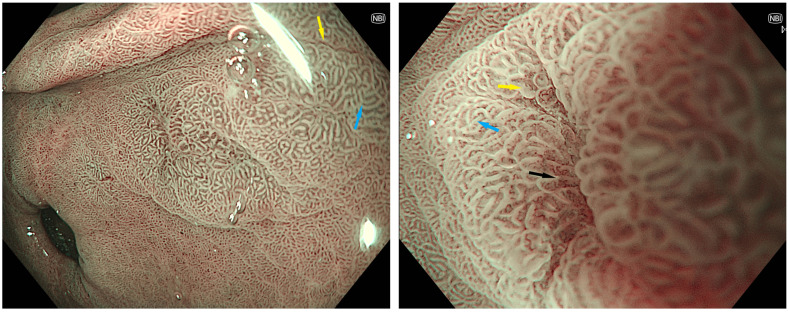
Endoscopic appearance of GIM with NBI. Non-magnification NBI (**left**) demonstrates a blue–whitish, tubulovillous pattern in the gastric antrum. A white turbid band (yellow arrow) and light blue crest (blue arrow) can be appreciated. Digital magnification (1.5×) with near-focus NBI (**right**) is focused on the region of GIM adjacent to an erosion (black arrow) with increased vascular prominence.

Because MTB and LBC are often best appreciated with magnification endoscopy, which has had limited availability until recently, particularly in the Western world [63], the use of image-enhanced endoscopy (IEE) has been investigated as a means of improving detection. Equipment-based IEE techniques, which do not need complex dyes, include narrow-band imaging (NBI), for which there is the most evidence, as well as flexible spectral imaging colour enhancement and i-SCAN. With standard magnification and NBI alone, a blue–whiteish area or tubulovillous pattern can be appreciated in GIM (Figure 2). Second-generation NBI consistently outperforms high-definition white-light endoscopy (HD-WLE) for the detection of GIM and dysplasia, where sensitivity has been reported in the range of 25–75% with a high specificity of >90% [64,65,66]. A meta-analysis published in 2023 by Rokkas and Ekmetzoglou reviewed five studies assessing NBI without magnification endoscopy for the detection of IM and reported a pooled sensitivity of 70% (from 55 to 82%) and specificity 94% (from 66 to 99%) with AUC 0.84; the sensitivity and AUC improved to 85% and 95% if magnification was used [67]. Further, NBI has a sensitivity of 87% and specificity of 97% for the detection of early cancerous lesions associated with an irregular and architecturally distorted mucosal and vascular appearance; sensitivity improves to >90% when magnification is used [68]. It is noteworthy that the definition of magnification endoscopy varies and can refer to a combined optical and digital magnification of 1.5×, or higher optical magnifications of up to 125–150×, with the latter generally requiring specialised endoscopes [63]. Rokkas and Ekmetzoglou did not divide these studies in their meta-analysis, but there is no obvious trend when comparing each study individually, and in a retrospective study with digital 1.5× magnification, which is found on most modern standard endoscopes, the sensitivity and specificity was 85% and 98%, respectively [69] (Table 1). Other forms of IEE, particularly blue laser imaging with magnification, have shown similar superior results compared to the use of white light alone [68,70]. IEE with magnification should, therefore, be a routine component of the assessment.

Other advanced endoscopic techniques, including confocal microscopy, chromoendoscopy with or without magnification, and Raman spectroscopy, have all shown very high sensitivity and specificity for the detection of IM with area under the curve >0.90, but their expense, complexity (adding to procedural time) and steep learning curve have limited their uptake and utility in clinical practice [71,72,73,74].

**Table 1:** Characteristics of the six studies [67] utilising magnification endoscopy with NBI for the identification of GIM.

Study	Participants	Study Design	Endoscope	Magnification	Sensitivity	Specificity
Uedo et al. (2006) [75]	107 Japan	Prospective	GIF-Q240Z	80× (optical)	89% (83–96%)	93% (88–97%)
An et al. (2012) [62]	47 South Korea	Prospective	GIF-H260Z	85× (optical)	72.1%	96.0%
Savarino et al. (2013) [76]	100 Italy	Prospective	GIF-Q160Z	115× (optical)	80% (67–92%)	96% (93–99%)
Ang et al. (2015) [65]	458 Asia Pacific	Prospective	GIF-290 and 190	Unclear	92.3% (80.6–97.5%)	94.3% (85.3–98.3%)
Drasovean et al. (2018) [77]	59 Romania	Prospective	HQ-190	150× (optical)	80.43% (70.9–88%)	80% (69.9–87.9%)
Sobrino-Cossio et al. (2018) [69]	338 Mexico	Retrospective	H-180	1.5× (digital)	85% (76.7–91.4%)	98% (96.7–98.9%)

There are two major schools of thought regarding the need for non-targeted mapping biopsies in addition to careful endoscopic assessment with the targeted biopsies of suspicious lesions outlined above.

Some advocate for mapping biopsies, generally applying the updated Sydney pro-tocol with samples from antrum greater and lesser curvatures, corpus greater and lesser curvatures, and the incisura [78]. As mentioned, a meta-analysis has demonstrated that standard IEE does not have 100% sensitivity for all IM or dysplasia [68]. In a prospective study by Buxbaum et al., 112 patients underwent HD-WLE and NBI examinations by two separate endoscopists, and subsequent targeted biopsies, followed by updated Sydney protocol mapping; a per-patient analysis showed that 100% of IM cases would have been detected by NBI with mapping, whereas 70.6% would have been seen on NBI with HD-WLE and 82.4% on HD-WLE with mapping [66]. Notably, NBI alone appeared to miss seven of eight cases of IM in the body, as seen on the mapping; it is unclear whether any cases would have been misclassified as limited rather than extensive IM. Further, in a comparative study of NBI-targeted biopsies and white light with mapping in 119 participants, dysplasia was found in 10% when using WLE and mapping, but was seen in 7% when using only NBI-targeted biopsy (*p* = 0.5); there was an additional case of gastric cancer that was only detected with WLE and mapping [79]. These data support the use of routinely updated Sydney protocol mapping in addition to NBI to increase yield.

However, some have pointed out heterogeneities in the study results, suggesting a learning curve, and that the above studies did not use magnification endoscopy; therefore, in experienced hands, mapping may be less beneficial at the expense of a longer procedure time and more biopsies [80]. Furthermore, it has not been clearly demonstrated that the additional findings from non-targeted biopsies change management or outcomes in most cases, as IM can be a patchy process and it is the overall impression which determines outcomes. Indeed, a more recent study in 95 patients where a non-expert endoscopist performed NBI-targeted biopsy in each of five regions of the stomach, or random biopsy if no targets could be identified, found that, even with a relatively inexperienced proceduralist (60 cases of IM only), the sensitivity for detecting extensive IM with magnification NBI-targeted biopsy was 88.4% (versus 100% with mapping), and the negative predictive value was 94.7% [81].

Overall, updated Sydney protocol mapping should be strongly considered where magnification endoscopy is unavailable, in low-to-moderate-incidence settings where experience with endoscopic techniques is lower, or in high-risk individuals.

To further address the concept of a global assessment of upper endoscopy as a risk predictor, whereby more extensive disease confers a more progressed state, the Endoscopic Grading of Gastric IM (EGGIM) scoring system was proposed in 2017 [82]. This involves the use of IEE to assess the same five regions of the stomach that are assessed with the updated Sydney protocol. For each region, extent is scored as 0 (no IM), 1 (≤30% IM) or 2 (>30% IM), giving a maximum score of 10. An EGGIM score ≥5 is considered higher risk, and a small 2022 meta-analysis of three studies demonstrated an odds ratio of 7.46 (95% CI 3.41 to 16.31) for early gastric neoplasia when compared with EGGIM 0–4 [83].

### 3.3. Histologic Features

Currently, histology remains an important tool for confirming a diagnosis of IM, excluding dysplasia or cancer, and as an adjunctive tool for the diagnosis of *H. pylori*. The Operative Link on Gastritis Assessment (OLGA) and Operative Link on Gastric Intestinal Metaplasia Assessment (OLGIM) systems were developed to grade and stage atrophic gastritis and GIM based on Sydney protocol biopsies [84,85]. In each, the severity of atrophy/IM in the antrum (including incisura) and body are combined to provide an overall staging (Table 2). The assessment and scoring of IM are generally more consistent than atrophic gastritis, with less interobserver variation [85].

Higher stage (OLGA/OLGIM III/IV) is associated with a higher risk of gastric cancer as compared with lower-stage atrophy/IM, as demonstrated in a meta-analysis published in 2018, where the odds ratios for gastric cancer for OLGA and OLGIM, respectively, were 2.64 (95% CI: 1.84–3.79) and 3.99 (95% CI: 3.05, 5.21). Importantly, EGGIM ≥5 has high concordance with OLGIM III/IV with an AUC of 0.97, reinforcing the role of endoscopic evaluation as a means of risk assessment [83].

However, OLGIM stage I/II still carries an increased risk of cancer. Marcos et al. found, in a case-control study, that OLGIM I/II was associated with an increased risk of early gastric neoplasia compared to no IM (adjusted odds ratio 11.5, 95% CI from 4.1 to 32.1) [86]. More recently, the Singapore Gastric Cancer Epidemiology and Molecular Genetics Programme (GCEP), a longitudinal, prospective study of 2980 patients undergoing screening gastroscopy (44.3% with IM), reported that, amongst patients with OLGIM II, the rate of early gastric neoplasia was 108.8/100,000 person–years, with a hazard ratio of 7.34 (95% CI 1.60 to 33.7) [53].

**Figure 3:**
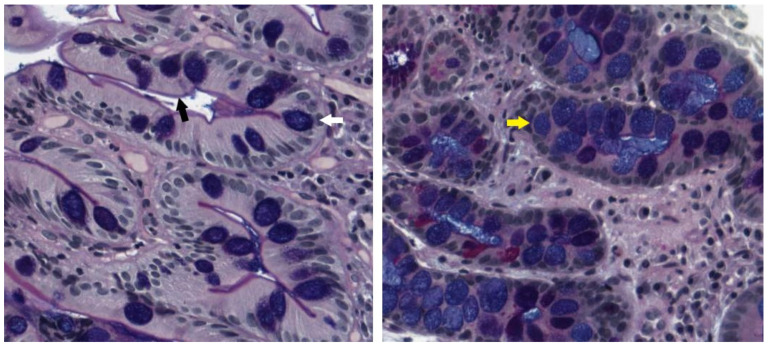
AB-PAS staining of GIM. Complete IM (**left**) with small intestinal epithelium characteristics such as brush border (black arrow) and goblet cells (white arrow). Incomplete IM (**right**) with colonic epithelium characteristics with no brush border and altered mucin staining (here, acidic, which appears in blue, denoted by the yellow arrow).

GIM has been further histologically classified as complete (type I) or incomplete (type II and III). Complete IM resembles small-intestinal epithelium with a brush border, Paneth cells, eosinophilic enterocytes and well-formed goblet cells. Incomplete IM adopts a more colonic-appearing epithelium, with no brush border, and irregular mucin droplets (Figure 3). Within incomplete IM, types II and III have been traditionally distinguished on high-iron diamine and Alcian-blue/periodic acid Schiff (AB-PAS) staining, with type II demonstrating sialomucins and type III predominantly showing sulfomucins [87]. Both complete and incomplete IM are associated with a gain in intestinal mucin (*MUC2*), but complete IM is generally associated with the loss of gastric mucin markers (*MUC1, MUC5AC* and *MUC6*), whereas in incomplete IM, these are frequently preserved [88,89]. This suggests a less differentiated phenotype in incomplete IM, which may confer a higher risk of premalignant change. In keeping with this, clinical studies have consistently shown that incomplete IM is associated with 3.7-fold risk of dysplasia compared to complete IM, and type 3 confers 2.9-fold higher risk than type 2 [90]. In clinical practice, distinctions between the type 2 and 3 IM are not routinely made, as high-iron diamine is no longer used in most laboratories due to concerns regarding its toxicity. The distinction between complete and incomplete IM can still be made based on morphology with routine haematoxylin-eosin staining [91]. However, although incomplete IM is a clear marker of risk, its inclusion in guidelines has been variable, in part because of concerns regarding its reproducibility between pathologists [92].

### 3.4. Current Guidelines

Reflecting the uncertainty surrounding the optimal management of GIM, recommendations from national societies in low-incidence countries are varied (Table 3). The North American association guidelines do not recommend routine surveillance of non-dysplastic GIM [93]; in contrast, the British Guidelines recommend either a thrice yearly surveillance for most cases, or no surveillance in cases with limited IM [92]. The European guidelines provide the most detailed recommendations for determining differing surveillance intervals, taking into account topography (extensive versus limited), family history and the persistence of *H. pylori*, the presence of ‘severe atrophy’ or incomplete IM and concurrent autoimmune gastritis, while not recommending surveillance for limited IM [94]. However, EGGIM or OLGA/OLGIM scoring are not routinely included, and some at-risk individuals (as previously discussed) may, therefore, be missed.

## 4. Future Directions

### 4.1. Approaches to Population-Level Screening and Surveillance

Many uncertainties remain in the risk stratification and management of GIM. At the population level, screening for GIM is not generally recommended in low-incidence countries as it is not thought to be cost-effective [95]. Several studies have shown cost-effectiveness in intermediate-risk settings such as in Singapore, particularly when gastroscopy is combined with same-day colonoscopy [96,97,98]. However, whether there are subgroups within low-incidence countries, such as ethnic groups with a higher incidence of IM and those with long-standing *H. pylori* infection, where screening should be considered (similar to the practice in screening for Barrett’s oesophagus), is still to be determined [99,100].

Furthermore, after GIM has been diagnosed, the current screening schedules (3 years in most guidelines) have not been tested with randomised prospective studies. Of note, in the longitudinal follow-up study of 1755 Italian patients who had undergone upper endoscopy for dyspepsia, the earliest gastric cancer occurred at 23 months in an OLGIM III patient [101]. In the Singaporean GCEP study, the median time to gastric neoplasia in the OLGIM III/IV group was 22.7 months (range 12.7–44.8 months) and in the OLGIM II group, it was 50.7 months (range 28.4–73.3) [53]. These two studies suggest that a shorter timeframe to follow-up may be required in high-risk individuals.

On the other hand, evidence supporting the practice of discontinuing surveillance in perceived ‘low risk’ (usually limited IM) patients is required. A dynamic change on follow-up endoscopy is one strong predictor of risk, which may be able to guide discharge from surveillance. Song et al. suggested that if any progression in IM was seen on follow-up endoscopy, the standardised incidence ratio of cancer was 30–40, whereas there was no significant difference in incidence for groups with non-progression or regression of IM [49].

### 4.2. Artificial Intelligence and Machine Learning

At the procedural level, artificial intelligence (AI) to enhance endoscopic diagnosis is likely to be the major advance in the coming decades and may help to reduce the heterogeneity seen in studies of endoscopic assessment. Two groups, using convolutional neural networks, have developed systems for detecting IM using magnification endoscopy IEE, yielding sensitivities of >90%, specificity 70–85% and accuracy ~90%, and outperforming endoscopists [102,103]. Models have also been developed to detect atrophic gastritis and active *H. pylori* infection, although the accuracy of the latter is slightly lower [104,105]. Evidence of endoscopic features that can distinguish complete and incomplete IM is currently lacking, but would be an area for further AI studies, given the prognostic significance of identifying incomplete IM.

### 4.3. Biomarkers

Although AI may reduce the need for biopsies to confirm diagnosis, tissue is likely to still play a role in the risk stratification and management of IM, as new histological and molecular markers are discovered and utilised. Given the concern regarding subjectivity in reporting complete and incomplete IM, there has been interest in tissue biomarkers, which may aid in subtyping.

*SLFN5* is expressed by T lymphocytes in gastric mucosa and is upregulated by inflammation. On immunohistochemical staining and subsequent scoring, the SLFN5 stromal score was found to be a significant risk factor for progression to both cancer (OR 18.1, 95% CI 4.14–79.1) and incomplete IM (OR 71.3, 95% CI 7.14–712.9).

*AQP3* is expressed on goblet cells in IM and was demonstrated to be associated with both increased severity and type III incomplete IM [106,107].

More recently, CD10, which is a brush border protein found only in small intestine (and not in colon) was shown to have excellent sensitivity and specificity for complete IM (87.5–94.9% and 97–100%, respectively) [108]; the same study found that Das1, an antibody to the colon epithelial protein, was associated with both incomplete IM and also with complete IM in patients with concurrent gastric cancer, and therefore may be a biomarker that can predict risk regardless of histological subtype.

More work is required to validate the findings with these four biomarkers in prospective cohorts and translate them into clinical practice.

Multiple non-invasive biomarkers have also been investigated as a means of screening for atrophy, IM, dysplasia or cancer, but few have progressed beyond the preclinical stage. Of these, the pepsinogen I/II ratio has been most widely investigated. This is based on the concept that pepsinogen I is secreted by fundic glands in the gastric fundus and body, while pepsinogen II is secreted by the entire stomach and duodenum; therefore, pepsinogen I and pepsinogen I/II ratio will fall with increasing inflammation/atrophy [109]. Serum gastrin, which is produced in antral G cells and therefore tends to rise with gastritis affecting the corpus and fall with antral-predominant atrophic gastritis, and *H. pylori* serology have been combined with the pepsinogen I/II ratio to improve performance [110,111,112]. While the results have been promising, the uptake of these tests in the clinical setting has been limited due to variability in the cut-offs, sensitivities and specificities of different assays [113].

### 4.4. Genomic, Epigenomic and Molecular Markers Uncover the Pathogenesis of IM and Stratify Risk

(Epi)genomic changes in IM were profiled to better understand the molecular drivers underpinning its pathogenesis, which may present future targets for intervention. Huang et al. profiled 138 cases of IM, as expected, revealing a higher mutation rate than normal controls (albeit much lower compared to non-hypermutated gastric cancer: 2.6 versus 6.9 mutations/Mb), with mutations in *FBXW7* (a tumour-suppressor gene) appearing to recur [114]. This is in keeping with the idea that the accumulation of genomic instability in the IM state predisposes to cancer. Amongst this cohort, shortened telomeres and chromosome 8q amplification were associated with subsequent dysplasia or cancer [114]. The same study showed that global DNA hypermethylation increased above normal samples, and at higher levels with more severe IM, while intragenic hypomethylation was a feature of gastric cancers.

In a more recent study by the same group, which is currently in pre-print without finalised peer-review (8 June 2023), 26 IM driver genes were identified. This included *SOX9* mutation (transcription factor involved in stem cell homeostasis), which was enriched in pre-cancerous lesions, whereas mutations in *ARID1A* (chromatin regulator), *KRAS* and *TP53* were enriched in gastric cancers [115]. By utilising spatiotemporal genomic profiling and transcriptomics, the proportion of intestinal lineage cells was demonstrated to increase in severe IM while gastric isthmus cells reduced, and gastric cancer cells most closely resembled intestinal stem cells. This supports the theory that the gastric stem cell undergoes genetic changes under stress, which gives rise to both IM and eventual cancer. One interesting theory raised by Huang et al. on the basis of their transcriptomic data, which may link multiple aspects of premaligant gastric lesions, is the possibility that spasmolytic peptide expressing mucosa (SPEM), an alternative premalignant lesion to gastric cancer initially described through animal studies [116], may be closely related to a subset of incomplete IM in the gastric corpus/cardia. Different populations of gastric epithelial stem cells have been described between the antrum, corpus isthmus and corpus base, characterised by different markers including Lgr5+, Mist1+ and Troy+ [31]; how each of these may give rise to different premalignant or malignant lesions is an area of ongoing research. Indeed, multiple foci of IM within a patient may arise from multiple different stem cells and exhibit different features.

A final area of emerging interest is the role of non-coding RNA, such as microRNA, which may modulate a range of cellular processes, and thereby predispose to or protect against malignancy [117]. Alterations in microRNA pattern are seen with *H. pylori* infection, as well as separate stages along the Correa cascade [118]. The serum level of the microRNA17-92 cluster has been proposed as a non-invasive test to predict intestinal metaplasia with an AUC of up to 0.996, sensitivity of 98% and specificity of 95% compared with normal controls, and an AUC of up to 0.988, sensitivity of 93% and specificity of 96% compared with gastric cancer [119]. This will require prospective validation but is a promising future biomarker and potential therapeutic target.

It is likely that integrating genomic and molecular data will allow for better risk stratification. A combined clinical–genomic model has been proposed, based on the GCEP data, which may outperform clinical-only models (AUC 0.846 versus AUC 0.707), but this will need to be validated in other cohorts [115].

### 4.5. Microbiome and the Immune Landscape

The possibility of dysbiosis serving as a contributor to IM and cancer pathogenesis has been raised [120,121]. Differing patterns in gastric microbiome diversity have been described in the premalignant versus malignant, and pre-*H. pylori* eradication versus post-eradication states, although there is significant heterogeneity in these findings [122,123,124]. Chronic inflammation again likely plays a role; Huang et al. identified, on transcriptomic data, a subset of IM found in the gastric body, which was associated with increased clusters of bacterial communities normally associated with the oral cavity and with higher inflammatory signatures [115]. The gastric microbiome presents a potential future therapeutic target in treatment or prevention, but substantial work is required to better understand the mechanisms of action and provide widely available testing in the clinical setting.

The immune landscape of IM likely plays a role in determining which cases progress, but there have been limited data to date. Some studies have shown reduced CD4 and CD8 T lymphocytes with advancements along the Correa cascade, suggestive of altered immune surveillance, but there have been variations in this trend [125,126,127]. Song et al. profiled the immune environment using CIBERSORT to show that the proportion of regulatory T lymphocytes and undifferentiated (M0) macrophages was higher than normal controls, while the proportion of CD4 memory T lymphocytes and dendritic cells was lower [128]. The interaction of these cells with each other and IM warrants further investigation, as well as the intersection with the specific immune response to *H. pylori* [129,130].

### 4.6. Utilising Integrated Variables to Increase Precision of Risk Prediction

**Figure 4:**
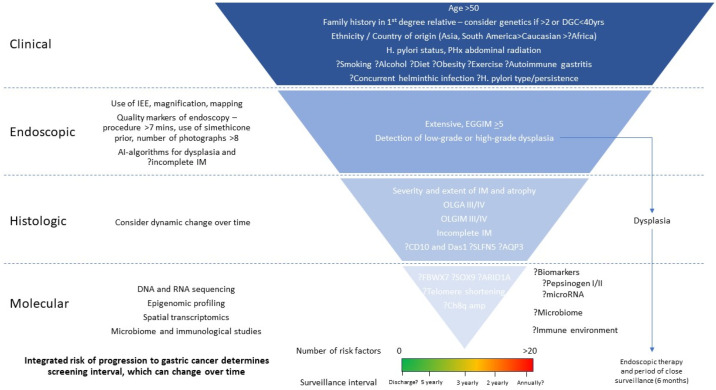
Proposed rubric for risk stratification of GIM. Currently, cumulative risk factors guide surveillance intervals. In future, an integrated risk score may allow for better precision prediction to guide surveillance.

In low-incidence countries, current guidelines for surveillance intervals for GIM consider, to varying degrees, a small number of clinical, endoscopic and histological risk factors (Table 3), with limited prospective studies to support current practices. These algorithms will become more accurate and refined with the increasing data available for the risk stratification of patients, as outlined above. One area of research will be the interaction between these various risk factors, and how they modulate an individual’s overall risk. An aspirational ideal is the development of risk calculators, using a rubric that can accurately integrate clinical, endoscopic, histological, and molecular risk factors to produce an overall risk score (Figure 4). Prospective studies will then be required to validate the shorter surveillance intervals for those at the highest risk, and longer intervals, or the even cessation of surveillance, in those at the lowest risk.

## 5. Conclusions

Gastric cancer will continue to be a cause of significant morbidity and mortality in the coming decades, particularly in view of the aging global population. While population-based screening has been a practical and successful approach in high-risk settings to improve outcomes, it will not be cost-effective or viable in lower-incidence populations. Therefore, focus has been placed on the detection of premalignant lesions, the most recognised and studied of which is GIM. However, only a small proportion of those with GIM will progress to dysplasia and cancer, and this risk is heterogeneous, representing a burden of oversurveillance to some individuals and the healthcare system. A few major risk factors were identified: advanced age, family history, probably non-Caucasian ethnicity and smoking, extensive topography, severe atrophy and incomplete IM on histology. Mapping biopsies are likely to continue to play a role in management, particularly as further immunohistochemistry, genomic studies, single-cell and spatial transcriptomics is being utilised to identify better predictors of risk. AI with deep convoluted neural networks is likely to improve detection, even amongst expert endoscopists, as has been demonstrated with the adenoma detection rate in colonoscopy [131]. Clinical studies are required to prospectively assess these risk factors in the real-world setting, and to optimise surveillance intervals (or, conversely, determine who can be safely discharged from follow-up).

## Figures and Tables

**Table 2:** OLGA and OLGIM staging systems.

		**CORPUS (BODY)**			
	Atrophy Score	No atrophy (0)	Mild atrophy (1)	Moderate atrophy (2)	Severe atrophy (3)
ANTRUM (INCLUDING INCISURA)	No atrophy (0)	Stage 0	Stage I	Stage II	Stage II
	Mild atrophy (1)	Stage I	Stage I	Stage II	Stage III
	Moderate atrophy (2)	Stage II	Stage II	Stage III	Stage IV
	Severe atrophy (3)	Stage III	Stage III	Stage IV	Stage IV
		**CORPUS (BODY)**			
	IM Score	No IM (0)	Mild IM (1)	Moderate IM (2)	Severe IM (3)
ANTRUM (INCLUDING INCISURA)	No IM (0)	Stage 0	Stage I	Stage II	Stage II
	Mild IM (1)	Stage I	Stage I	Stage II	Stage III
	Moderate IM (2)	Stage II	Stage II	Stage III	Stage IV
	Severe IM (3)	Stage III	Stage III	Stage IV	Stage IV

**Table 3:** Comparison of gastric IM guidelines from European Society of Gastrointestinal Endoscopy (ESGE), British Society Guidelines (BSG) and American Gastroenterology Association (ACG).

	ESGE (2019)	BSG (2019)	AGA (2020)
Extensive IM	Thrice yearly	Thrice yearly	Routine surveillance not recommended. Patients with GIM at higher risk for gastric cancer who place high value on potential but uncertain reductions in gastric cancer mortality, and who place a low value on the potential risks of surveillance endoscopies, may reasonably elect for surveillance. Consider surveillance 3–5 times a year.
Limited IM, no risk factors	No surveillance	No surveillance	
Limited IM, family history OR incomplete or persistent H pylori	Thrice yearly	Thrice yearly	
Limited IM, severe atrophy	Thrice yearly	Thrice yearly	
Limited IM, severe atrophy, family history	Considered 1–2 times a year	Thrice yearly	
Autoimmune gastritis	Considered 3–5 times a year		

## Data Availability

Not applicable.
